# Biomarkers of Inflammation, Immunosuppression and Stress Are Revealed by Metabolomic Profiling of Tuberculosis Patients

**DOI:** 10.1371/journal.pone.0040221

**Published:** 2012-07-23

**Authors:** January Weiner, Shreemanta K. Parida, Jeroen Maertzdorf, Gillian F. Black, Dirk Repsilber, Anna Telaar, Robert P. Mohney, Cordelia Arndt-Sullivan, Christian A. Ganoza, Kellen C. Faé, Gerhard Walzl, Stefan H. E. Kaufmann

**Affiliations:** 1 Department of Immunology, Max Planck Institute for Infection Biology, Berlin, Germany; 2 Department of Biomedical Sciences, University of Stellenbosch, Cape Town, South Africa; 3 Biomathematics/Bioinformatics Group, Genetics and Biometry, Leibniz Institute for Farm Animal Biology, FBN, Dummerstorf, Germany; 4 Metabolon, Inc., Durham, North Carolina, United States of America; Institute of Infectious Diseases and Molecular Medicine, South Africa

## Abstract

Although tuberculosis (TB) causes more deaths than any other pathogen, most infected individuals harbor the pathogen without signs of disease. We explored the metabolome of >400 small molecules in serum of uninfected individuals, latently infected healthy individuals and patients with active TB. We identified changes in amino acid, lipid and nucleotide metabolism pathways, providing evidence for anti-inflammatory metabolomic changes in TB. Metabolic profiles indicate increased activity of indoleamine 2,3 dioxygenase 1 (IDO1), decreased phospholipase activity, increased abundance of adenosine metabolism products, as well as indicators of fibrotic lesions in active disease as compared to latent infection. Consistent with our predictions, we experimentally demonstrate TB-induced IDO1 activity. Furthermore, we demonstrate a link between metabolic profiles and cytokine signaling. Finally, we show that 20 metabolites are sufficient for robust discrimination of TB patients from healthy individuals. Our results provide specific insights into the biology of TB and pave the way for the rational development of metabolic biomarkers for TB.

## Introduction

Metabolomics provides unprecedented insights into the biology of an organism in the state of disease. In particular, metabolites can be viewed as a close recapitulation of the disease phenotype, closer to ongoing pathogenesis in an organism than changes in gene expression [1]. Consequently, metabolomics has deepened our understanding of biological mechanisms involved in several noninfectious diseases and provided a platform for the identification of new biomarkers [2]. Metabolic signatures have proven their value in several diseases, such as Alzheimer’s disease [3], Parkinson’s disease [4], myocardial ischemia [5], hypertension [6], cancer [7] and diabetes [8]–[11]. In contrast, fewer studies have specifically addressed the metabolomic alterations that occur in infectious diseases [12]–[16].

Tuberculosis (TB) is caused by the intracellular bacterial pathogen *Mycobacterium tuberculosis* [17], [18]. It is characterized by chronic latency without clinical symptoms in the vast majority of infected individuals. Of the more than 2 billion people infected with *M. tuberculosis* globally, less than one-tenth is likely to develop active TB during their lifetime and in the majority of cases infection remains in an asymptomatic stage of latency. Even though TB can be cured, drug treatment is long-lasting and complex, and requires at least three drugs given over a minimum of 6 months. In many regions, notably in developing countries with high TB incidences, diagnosis is neither sensitive nor specific, and an estimated 40% of TB patients fail to be correctly diagnosed [19]. The tuberculin skin test (TST) diagnoses immunologic sensitization to mycobacteria, indicating exposure, and hence cannot be used to distinguish infected healthy individuals from patients with active TB. More recently, gene expression profiling has shown promise in differentiating between infection states in TB [20]–[22].

We investigated the feasibility of identifying small molecule biochemical profiles in serum for gaining novel biological insights into the mechanisms underlying TB. The aim of this study is to use metabolites as biomarkers of TB infection and disease status, and to elucidate their role in protection and pathogenesis in TB. We acquired information on 428 distinct small molecules, including amino acids, short peptides, carbohydrates, fatty acids, nucleotides and cofactors, and compared the characteristic metabolomic profiles of three groups: (i) healthy *M. tuberculosis*-uninfected controls (TST^–^), (ii) latently *M. tuberculosis*-infected healthy individuals (TST^+^), and (iii) patients with active TB (TB^active^; see Table S1).

We defined several remarkable differences between the metabolic profiles of healthy subjects and TB patients, including lower relative abundances of amino acids, medium-chain fatty acids and lysophosphatidylcholines (LPCs), higher relative abundances of fibrinopeptides and adenosine degradation products inosine, hypoxanthine and ribose, as well other compounds, such as bile acids and uremic toxins. The observed differences and correlations between compound abundances allowed us to construct functional clusters and infer their biological significance in TB disease progression. In addition, we could identify a serum metabolomic biomarker set that distinguishes active TB from the two groups of healthy subjects, which is a necessary first step towards the use of metabolic biomarkers in TB diagnosis. We also found that distinct cytokine abundances in several instances correlated with the relative abundances of metabolites, indicating a functional link between these systems. Finally, we studied the role of tryptophan degradation by indoleamine 2,3 dioxygenase 1 (IDO1), a well-known immunosuppressive mechanism. We revealed that IDO1 expression was upregulated in pulmonary lesions from mice suffering from experimental TB. Moreover, *in vitro* infection of macrophages and dendritic cells (DCs) with *M. tuberculosis* mirrored findings observed in serum.

## Results

### Test Groups Differ in the Relative Abundance of Several Metabolites

**Figure 1:**
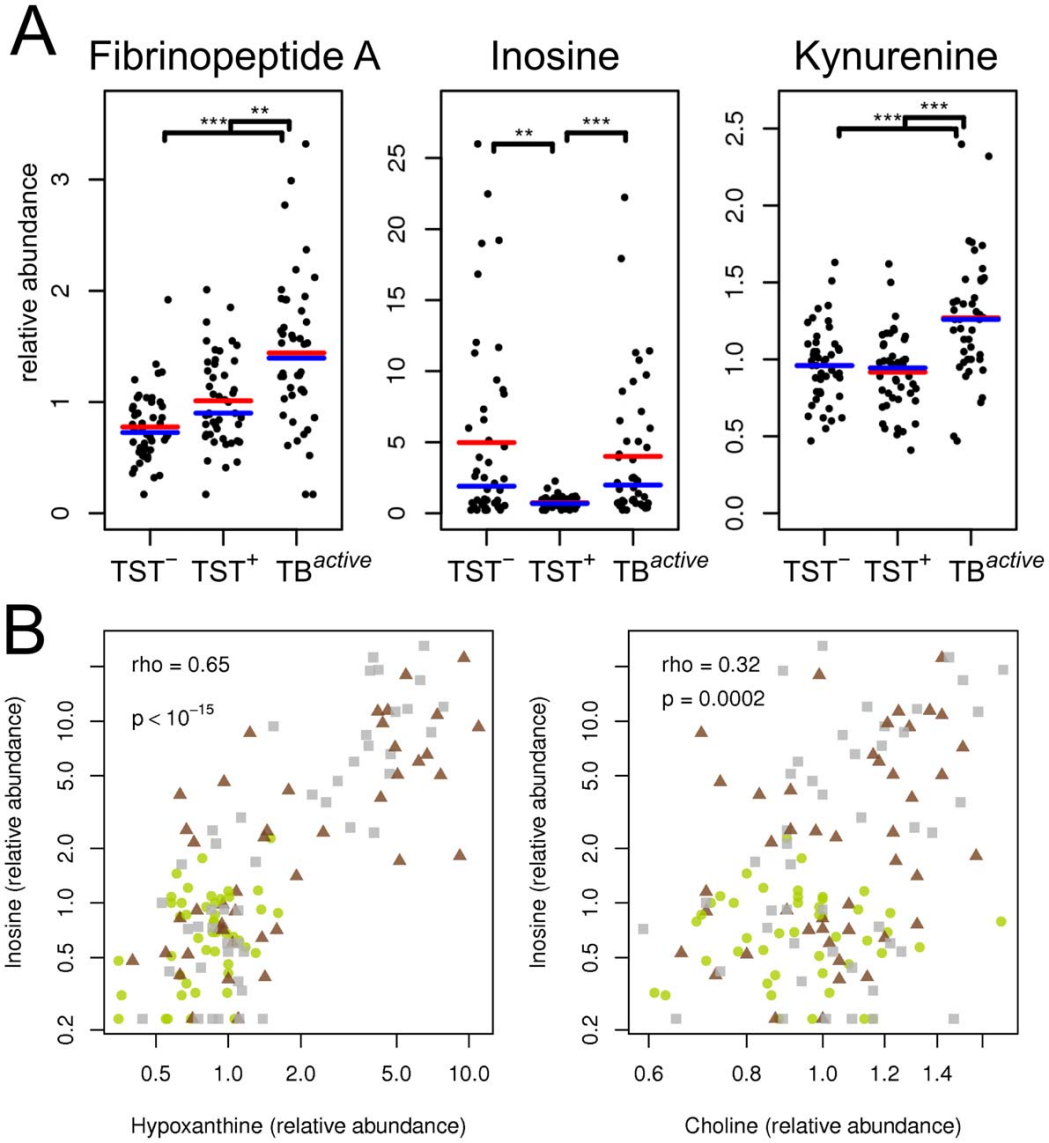
Examples of metabolite patterns in tuberculosis patients (TB^active^), healthy uninfected (TST^–^) and latently infected (TST^+^) individuals. (A) Changes in relative abundance of three exemplary small metabolites: fibrinopeptide A, inosine and kynurenine. Red line indicates sample mean, blue line indicates sample median. Stars indicate significant differences between profiles (result from t-test corrected for multiple testing; *, p<0.05; **, p<0.01; ***, p<0.001) (B) Correlation between abundance of inosine and abundance of hypoxanthine and choline. Colors and symbols denote study groups: grey squares, TST^–^; green circles, TST^+^; red triangles, TB^active^. Spearman correlation coefficients (rho) and corresponding p-values are given.

Differences in the abundance of small molecules in serum between the three study groups were determined by applying a t-test for each of the three possible comparisons (TST^–^ vs. TST^+^, TST^–^ vs. TB^active^ and TST^+^ vs. TB^active^), corrected for multiple testing at a significance threshold of 0.05. Results revealed significant differences for 176 compounds between the TB^active^ group and the two healthy groups (TST^–^ and TST^+^; see Table S2 and Figure 1).

**Figure 2:**
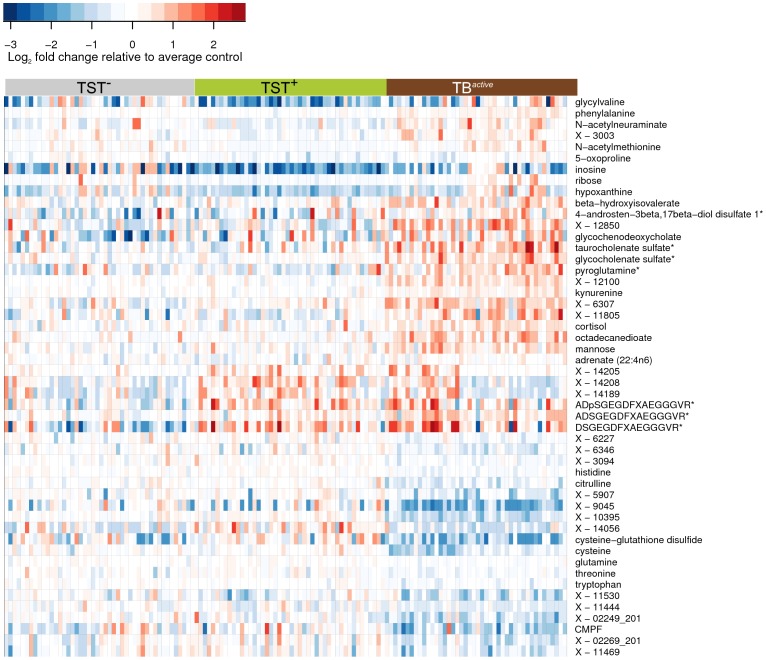
Heat map showing fold changes of small metabolic compounds in the three study groups, TB patients, healthy uninfected and latently infected individuals. Fold changes are relative to the average abundance in the TST^–^ group. Red indicates relative abundance higher than average in the TST^–^ group; blue indicates relative abundance lower than average in the TST^–^ group. Horizontal axis: samples belonging to the three study groups; vertical axis: top 50 compounds selected by variable importance in RF analysis, including compounds that could not be identified, but were strong predictors of sample status. Color bars above the heat map denote study groups: grey, TST^–^; green, TST^+^; red, TB^active^. See also Figure S2.

Among various other compounds, we found that several amino acids, such as histidine, cysteine, glutamine, tryptophan, citrulline and creatine, were at lower levels in the TB^active^ group compared to the two control groups, and only some amino acids were increased in abundance (including kynurenine, phenylalanine and pyroglutamine) in the TB^active^ group. Other identified compounds with markedly differentiated abundance between TST^+^ and TB^active^ groups were sialic acid (N-acetylneuraminate), 3-carboxy-4-methyl-5-propyl-2-furanpropanoic acid (CMPF), inosine, xanthine, hypoxanthine, fibrinopeptide A, glucose, and mannose (Figure 2 and Table S2). Intriguingly, we also identified six metabolites that differed significantly between the two healthy control groups, TST^–^ and TST^+^ (inosine, hypoxanthine, glycylvaline, 5-oxoproline and two unidentified compounds; see Table S2, A and Discussion).

### Differences in Small Metabolites can be Used as Specific and Sensitive Biosignatures

To determine whether differences in metabolite abundance between any two of the three study groups could be efficiently exploited for building a sensitive biosignature of TB status, we applied random forests (RF) classification, a supervised machine learning algorithm [23]. The TB^active^ group could be sensitively and specifically distinguished from the other two groups, TST^–^ and TST^+^ with a cross-validation error rate of 3.3%. Similar results were obtained for classification between the TB^active^ group and each of the healthy groups separately. Classification between TST^–^ and TST^+^ groups was error prone (i.e., overall cross-validation error rate of 41.3% and 32.6% for the two groups, respectively), due to highly similar metabolite profiles in these two groups of healthy individuals. Additional analyses revealed that less than 20 compounds sufficed for robust classification of TB^active^ with a cross-validation error rate of approximately 3% (Figures S1 and S2; Tables 1, 2 and S3). For illustration purposes, 10 samples from the TST^+^ and TB^active^ groups were randomly assigned to a test set, and the remaining training set was used to repeat all calculations. As expected from error rates calculated by an external cross-validation procedure, no misclassifications occurred in the resulting data set (Figure S2).

**Table 1:** Confusion matrices showing the results of external cross-validation for random forests models of the discrimination procedure among the tuberculosis patients (TB^active^) and healthy uninfected (TST^–^) and latently infected (TST^+^) individuals using the full set of metabolites.

Comparison	Factual class	Model prediction			% Error
TST^–^ vs TB^active^		TST^–^	TB^active^		
	TST^–^	45	1		2.20
	TB^active^	2	42		4.50
TST^+^ vs TB^active^		TST^+^	TB^active^		
	TST^+^	45	1		2.20
	TB^active^	1	43		2.30
TST^–^, TST^+^ and TB^active^		TST^–^	TST^+^	TB^active^	
	TST^–^	28	16	2	39.00
	TST^+^	13	32	1	30.00
	TB^active^	1	2	41	7.00

Each of the three matrices shows the error rates in one classification model: A, TST^–^ versus TB^active^; B, TST^+^ versus TB^active^; C, classification model for all three study groups. The values in each row indicate how many of the samples from a given factual group were correctly classified by the model in an external cross-validation bootstrapping procedure, and the respective error rates (%). See also Figures S1 and S2.

**Table 2:** The top 20 named metabolites identified in the random forests (RF) analysis as most important for discrimination between the TST^+^ and TB^active^ groups.

Biochemical name	Pathway	TST^–^	TST^+^	TB^active^
Histidine	Histidine metabolism	1.11	1.14	0.79
Cysteine	Cysteine, methionine, SAM and taurine metabolism	1.16	1.18	0.62
Threonine	Glycine, serine and threonine metabolism	1.14	1.24	0.85
Citrulline	Urea cycle; arginine and proline metabolism	1.14	1.14	0.70
Cysteine-glutathione disulfide	Glutathione metabolism	1.19	1.64	0.49
N-acetylneuraminate	Aminosugars metabolism	1.16	0.84	1.81
Glycocholenate sulfate*	Bile acid metabolism	0.94	0.94	1.99
Inosine	Purine metabolism, (hypo)xanthine/inosine containing	4.98	0.75	4.01
Tryptophan	Tryptophan metabolism	1.07	1.06	0.78
Mannose	Fructose, mannose, galactose, starch, and sucrose metabolism	0.85	0.93	1.53
3-carboxy-4-methyl-5-propyl-2-furanpropanoate (CMPF)	Fatty acid, dicarboxylate	1.58	1.68	0.77
Phenylalanine	Phenylalanine and tyrosine metabolism	1.00	0.92	1.31
Pyroglutamine*	Glutamate metabolism	1.07	1.06	2.13
Taurocholenate sulfate*	Bile acid metabolism	0.93	1.04	2.63
Glutamine	Glutamate metabolism	1.05	1.11	0.86
Octadecanedioate	Fatty acid, dicarboxylate	0.58	0.59	1.26
Urea	Urea cycle; arginine and proline, metabolism	1.14	1.22	0.88
Gamma-glutamylglutamine	g-glutamyl	0.93	1.25	0.63
Glycylvaline	Dipeptide	1.97	0.67	2.40
Aspartate	Alanine and aspartate metabolism	1.34	0.89	1.69

RF analysis indicates that 20 variables are sufficient for a robust classification. For a complete list including the compounds that could not be uniquely identified, see Table S2. Numbers correspond to the relative abundance of a given metabolite in the respective group. The significance of differences in abundances is shown in Table S1.

Metabolomic signatures that correctly classified TB^active^ patients comprised distinct clusters, which provided deeper insights into pathogenic mechanisms of TB. We applied hierarchical clustering with correlations between profiles as a distance measure, and bootstrapping for statistical assessment of the quality of clustering. This method created a tree of metabolites, and at each tree node a p-value was obtained by bootstrapping. Consequently, statistical significance could be evaluated for each potential cluster. Using a p-value threshold of 0.05, we found 18 clusters with at least five compounds. Among these, 12 clusters were of biological interest as they contained at least one compound showing significant differences between the three study groups (Table S2). Similar results were obtained using a substantially different method, sparse PLS discriminant analysis (SPLS-DA) [24].

### Biological Significance of Discriminative Clusters

**Figure 3:**
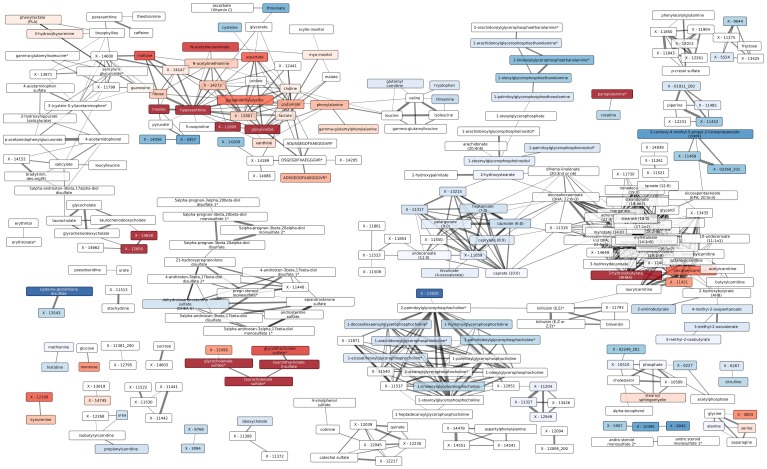
Network showing functional relationships between the small metabolic compounds in TB patients, healthy uninfected and latently infected individuals. Nodes correspond to metabolites; edges correspond to statistically significant correlation between residual small metabolite profiles corrected for study classes. Colors correspond to differences between the TST^+^ and classes (see Figure S3 for additional comparisons). Color intensity indicates significance of difference with darker colors corresponding to more significant differences. Metabolites with adjusted p value >0.05 are not colored. Line thickness corresponds to the absolute Spearman correlation coefficients corrected for groups (see Methods). See also Figure S3.

To understand the relationships between and within clusters, as well as to visually explore the underlying biological mechanisms, we constructed a network based on partial correlations between compounds. Group effects were removed such that compounds showing similar differences between groups, but no within-group correlation that would indicate a functional relationship, were not clustered together. For each compound, we fitted a linear model using the three study groups as a predictor variable and then used the residuals from this model to calculate the correlations between compounds. Based on these correlations, we constructed a network graph that can be explored visually (Figures 3 and S3).

In a first step, we validated our approach to identify functionally related compounds. We predicted that tightly coregulated compounds (e.g., a class of fatty acids) would form significant clusters. Consistent with this prediction, numerous groups of related compounds were reliably identified and grouped in tight clusters. For example, all medium-chain fatty acids (MCFAs) formed a uniform group of correlated biochemical compound profiles. Moreover, correct identification of functionally related compounds should allow close correlation of xenobiotics with their metabolites. Consistent with this prediction, degradation products of xenobiotics, including caffeine, stachydrine, salicylate and acetaminophen (Tylenol), were tightly correlated (Figures 3 and S3).

We labeled the clusters by the predominant pathway or type of metabolic compound found within the particular cluster (Table S4). Several of the clusters we identified included metabolic compounds that significantly differed between the study groups. Notably, we determined alterations in clusters of the following: amino acids, medium-chain fatty acids, carnitines, LPCs and fibrinopeptides. We did not find significant differences in the clusters of hormones, xenobiotics or long chain fatty acids.

### Abundance of Metabolites is Linked to Immune Response

**Figure 4:**
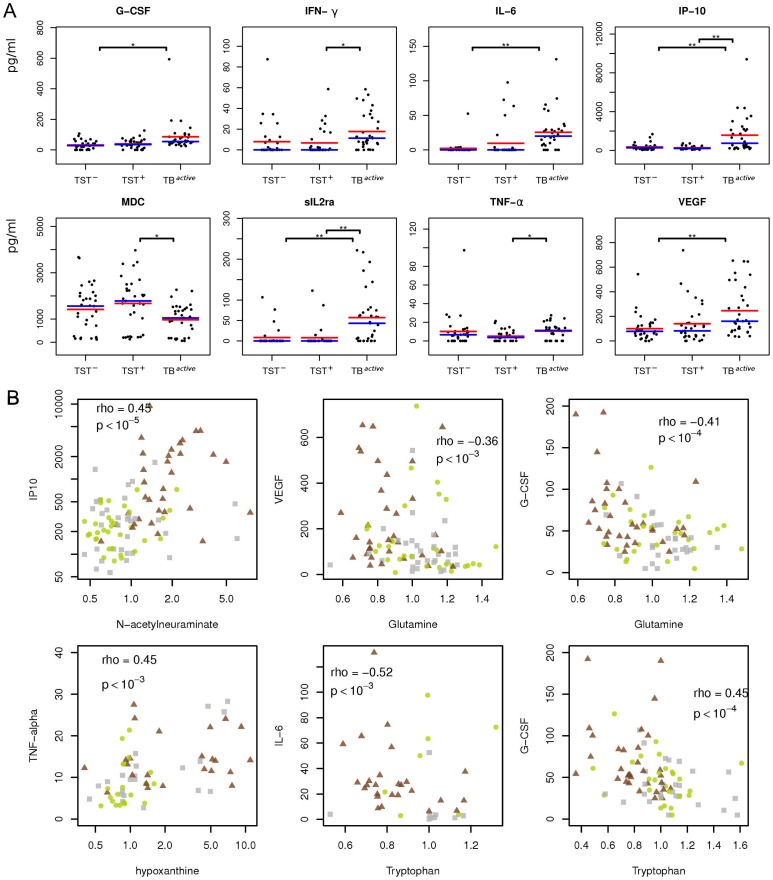
Abundance of cytokines and their correlation with selected metabolites in TB patients, healthy uninfected and latently infected individuals. (A) Strip charts showing abundances of eight cytokines that differed significantly between the study groups. Significance thresholds for a two-tailed t-test corrected for multiple testing: *, p<0.05; **, p<0.01; ***, p<0.001. Blue line indicates group median, red line indicates group mean. (B) Correlations between metabolic compounds and cytokines. Grey squares, healthy controls (TST^–^); green circles, latently infected individuals (TST^+^); brown triangles, active TB patients (TB^active^). Spearman correlation coefficient (rho) and p-values are given.

To determine whether there is a direct functional connection between serum abundance of metabolites and of immune mediators, we analyzed 35 cytokines/chemokines in 99 out of the 136 serum samples (note: sample number was reduced due to limited availability of aliquots). Significantly elevated serum concentrations of granulocyte colony-stimulating factor (G-CSF), interferon-gamma (IFN-γ), interleukin 6 (IL-6), C-X-C motif chemokine 10 (CXCL-10), soluble alpha chain of the IL-2 receptor alpha (sIL2ra) and vascular endothelial growth factor (VEGF), as well as lower concentrations of macrophage-derived cytokine (MDC) were detected in TB^active^ patients as compared to the two healthy groups (Figure 4). Notably, several of the differentially abundant cytokines in the study anticorrelated significantly with serum metabolites. For example, IL-6, IP-10 and sIL2ra were significantly correlated with the amino acids tryptophan and glutamine (p<10^−5^, r < –0.3). In total, out of 15,408 correlations between cytokines/chemokines and metabolic compounds tested, 211 significant correlations (p<0.05) remained after correction for multiple testing. We wanted to determine categories of metabolic compounds that frequently correlated with the abundances of cytokines and chemokines. To this end, we performed an enrichment analysis, which revealed significant enrichment of amino acids (p<10^–6^) and carbohydrates (p = 0.004). Therefore, this establishes a link between changes in the metabolic profile and the cytokine response in TB.

### Increased IDO1 Activity and Kynurenine Production

Sera of TB patients were characterized by a significant increase in the abundance of kynurenine, a product of tryptophan metabolism. The enzyme IDO1 catalyzes the degradation of tryptophan to kynurenine, with well-established immunosuppressive effects in mammalian pregnancy [25], [26], tumor resistance [27], [28], chronic infection [29]–[33] and autoimmune diseases [34]. Furthermore, IDO1 is critically involved in CD4 and CD8 effector T cell suppression, as well as in generation and activation of regulatory T cells [35], [36]. Therefore, we decided to investigate the role of IDO and its metabolites in immunity against TB.

**Figure 5:**
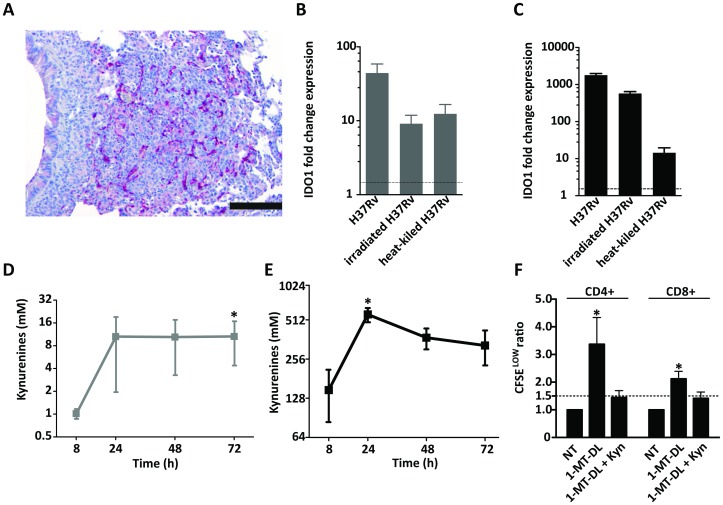
Demonstration of IDO1 expression and kynurenine production in response to *M. tuberculosis* infection and regulation of *M. tuberculosis*-specific T-cell responses by kynurenines. (**A**) Immunohistochemistry staining of formalin-fixed, paraffin-embedded tissue of a murine pulmonary TB lesion stained with anti-IDO polyclonal antibody; staining representative of lesions from five animals. Bar is equal to 200 nm. Human monocyte-derived dendritic cells (DCs) (**B**) and macrophages (**C**) were infected with *M. tuberculosis* H37Rv or stimulated with irradiated and heat-killed *M. tuberculosis* H37Rv for 24 h and indoleamine 2,3 dioxygenase 1 (IDO1) gene expression was measured by qPCR. Mean and standard deviation (SD) of fold-change IDO1 gene expression of one donor representative of four. Line indicates minimal significant fold change threshold equal to 1.5. DCs (**D**) and macrophages (**E**) were infected with *M. tuberculosis* H37Rv and cell culture supernatants were collected at indicated times for measurement of kynurenines by HPLC; kynurenine levels from uninfected controls were subtracted. Means and SD of four donors are depicted (C and D). Star indicates significance (p<0.05) in Friedman test.(**F**) Human monocyte-derived DCs were pulsed with purified protein derivative (PPD) and mannosylated lipoarabinomannan (ManLAM) and co-cultured for 4 days with autologous CFSE-labeled purified T cells (DC:T cell ratio 1∶20) in the presence or absence of 1-methyl-DL-tryptophan (1-MT-DL) and 3-OH-kynurenine (Kyn). Cell proliferation was assessed by CFSE dilution using flowcytometry. Figure representative of three independent experiments (ANOVA F = 4.1 for CD^+^CD4^+^ cells and F = 3.3 for CD3^+^CD8^+^ cells, p<0.05).

Consistent with elevated tryptophan degradation to kynurenine in TB, IDO1 protein was highly expressed in pulmonary lesions in mice suffering from experimental TB (Figure 5A). Human monocyte-derived DCs and macrophages strongly upregulated the expression of IDO1 after infection with *M. tuberculosis*; live bacteria induced stronger expression compared to heat-killed or irradiated bacteria (Figure 5B and C). In agreement with this finding, the enzymatic activity of IDO1, measured by the conversion of tryptophan to kynurenines by high-performance liquid chromatography (HPLC), was increased in infected host cells (Figure 5D and E). T cells stimulated with DCs pulsed with purified protein derivative (PPD) plus mycobacterial lipid (mannosylated lipoarabinomannan, ManLAM) and treated with the specific IDO1 inhibitor 1-methyl-DL-tryptophan (1-MT-DL) showed elevated proliferation compared to cells treated only with PPD and ManLAM. However, addition of kynurenine reversed this effect (Figure 5F), emphasizing the regulation of T cells by kynurenines.

## Discussion

Our analysis of several hundred serum small molecules has provided intriguing insights into the metabolomic status of three groups in *M. tuberculosis* infection: no *M. tuberculosis* infection (TST^–^); *M. tuberculosis* latent infection (TST^+^); and active TB disease (TST^active^). These metabolomic characteristics could be translated into a custom-made biosignature capable of discriminating healthy individuals with and without *M. tuberculosis* infection from TB patients.

In two recent studies, tissues from mice infected with *M. tuberculosis* [16] and lung granulomas from *M. tuberculosis*-infected guinea pigs [15] were profiled for metabolites using nuclear magnetic resonance (NMR). Although the number of metabolites profiled in these studies was significantly lower, several findings of these studies are congruent with our findings.

We applied a clustering procedure for groups of compounds that were functionally related. In each group, the compounds were significantly correlated, indicating their codependence on a specific metabolic process or systemic response. Consistent with this notion, several compounds expected to be present in a given group were significantly correlated. This procedure allowed us to link additional compounds that were not predicted to form a cluster. Notably, in many cases, differential abundance of serum metabolites between the different study groups could not be directly attributed to specific metabolic pathways. The presence of metabolic compounds in serum is rarely a simple consequence of metabolic processes within cells, but rather the result of a complex interplay between different parameters, such as unique metabolic and secretory features of cells in different organs. In the following, we discuss the most prominent metabolite clusters in the context of their biological relevance and their potential as custom-tailored biosignatures for TB.

### Tryptophan and Kynurenine

In the TB^active^ group, but not in the TST^−/+^ groups, kynurenine and its derivative, 3-hydroxykynurenine were elevated. The activity of IDO1, an enzyme that converts tryptophan to kynurenine, is increased under various inflammatory conditions resulting in heightened abundance of kynurenines [37]. Therefore, increased serum concentrations of kynurenines suggest upregulation or increased IDO1 activity in the TB^active^ group.

We tested the hypothesis that increased IDO1 activity is a direct consequence of stimulation of immune cells by *M. tuberculosis* and that this increased enzymatic activity leads to an increased level of kynurenines. We identified IDO1 in situ in granulomatous lesions obtained from the lungs of mice suffering from experimental TB. Furthermore, we observed elevated levels of IDO gene expression and enzymatic activity in human DCs and macrophages upon infection with *M. tuberculosis* (Figure 5B–E). Finally, we demonstrate human T cell regulation by kynurenines in TB (Figure 5F). These findings support the immune regulatory function of the active catabolites generated by the increased degradation of tryptophan during TB, suggesting that the tryptophan degradation pathway controlled by IDO1 is involved in the pathogenesis of this disease.

Increased abundance of kynurenine in serum from the TB^active^ group was significantly correlated with similarly increased abundance of the immunosuppressive stress hormone cortisol (p<10^–5^, r = 0.38). Glucocorticoids can activate IDO1 via glucocorticoid-induced tumor necrosis factor receptor (GITR) and protect against allergic bronchopulmonary aspergillosis [38]. Hence, the tryptophan catabolism pathway could be a mechanism by which corticoids exert immunosuppression [38]. Independently, in a recent publication, Suzuki et al. [39] found that serum IDO activity and kynurenine abundance is higher in TB patients.

### Fibrinopeptide A

Three forms of fibrinopeptide A were significantly correlated and elevated in both TST^+^ and TB^active^ groups with highest abundance in TB patients. Tuberculous granulomas are walled off from the surrounding tissue by a fibrinous wall. Fibrin deposition in granulomas resulting in release of fibrinopeptides could explain the high serum abundance of fibrinopeptides in TB patients and latently infected individuals. Interestingly, in female members of the study groups, another short peptide corresponding to a fragment of the C3 complement component was correlated with fibrinopeptide A and found at significantly higher serum concentrations in TB^active^ than in TST^–^ and TST^+^ groups.

### LPCs

One of the most prominent clusters in our analysis consisted of different LPCs, which were found at lower levels in the TB^active^ group but did not significantly differ between TST^–^ and TST^+^ groups. A major source of LPCs is the proinflammatory phospholipase A2, as plasma concentrations of LPCs significantly correlate with the activity of this enzyme [40]. Lower levels of LPCs have been observed in some types of cancer [41], [42], as well as in sepsis patients in whom lower LPC concentrations correlate with mortality [43]. Lower abundance of LPCs in TB patients could be mechanistically related to the finding that *M. tuberculosis* can induce macrophage apoptosis by inhibition of phospholipase A2 [44]. In contrast, Somashekar et al. [15] found a signature for lipolysis in the lung granulomas from *M. tuberculosis*-infected guinea pigs.

### Hypoxia Cluster

Inosine, hypoxanthine, ribose and xanthine formed one cluster, in which the compounds were present in high abundance in several individuals belonging to the TST^–^ and TB^active^ groups, and uniformly low in abundance in the TST^+^ group (Figure 1B). These compounds are part of the inosine breakdown pathway in which inosine is processed to hypoxanthine and ribose by the 5′ nucleosidase, and hypoxanthine is subsequently oxidized to xanthine by xanthine oxidase. Inosine, hypoxanthine and ribose have been defined as biomarkers of hypoxia, hypoxemia and ischemic brain injury [45], [46]. Inosine is a natural analog of adenosine and binds adenosine receptors A2A and A3 [47], [48], thus decelerating inflammation.

In tuberculous granulomas, hypoxia prevails and is thought to contribute to the containment of *M. tuberculosis*. An abundance of inosine catabolites in serum from the TB^active^ group is consistent with this notion. Reasons for higher abundance of hypoxia biomarkers in serum of the TST^–^ group as compared to the TST^+^ group remain to be clarified. A high prevalence of current and previous smoking was previously reported in the study community [49]. Uniformly low serum concentrations of breakdown products of inosine in the TST^+^ group argues against a major role of hypoxia in latent infection. Rather, since individuals with higher abundance of inosine are absent from the TST^+^ group, but present in the TST^–^ group, it is tempting to speculate that the occurrence of hypoxia is coincident with progression from latent infection to active TB disease. Notably, elevated serum levels of hypoxanthine have been also reported in severe childhood pneumonia [12]. Also, a hypoxia signature has been identified in lung granulomas of guinea pigs infected with *M. tuberculosis* [15] as well as in the organs of *M. tuberculosis*-infected mice [16].

Several other metabolites were correlated within the inosine pathway cluster. Methionine was present in serum at low abundance in the TB^active^ group. The biochemical derivative of methionine, N-acetyl-L-methionine (NAM), was detected in more than one-third of both of the TB^active^ and TST^–^ groups, but was undetectable in the TST^+^ group. The levels of methionine and NAM were inversely correlated. Although the physiological role of NAM during infection remains unclear [50], a relevant enzyme, methionine adenosyltransferase, is known to be regulated by hypoxia [51]. Choline abundance correlates significantly with abundance of inosine and xanthine, and is known to be a reliable biomarker in ischemia and acute coronary syndrome, released in the course of ischemic membrane damage and the activity of phopholipase D [37]. Lactate was found at higher levels in the TB*^active^* group, coinciding with a previous finding in mice and possibly related to increased glycolysis in granulomatous inflammation [16].

### MCFAs

Eight to twelve carbon-containing MCFAs are absorbed into the bloodstream and primarily metabolized in the liver. MCFA serum concentrations were significantly reduced in the TB^active^ group. Medium-chain triglycerides inhibit free radical formation and tumor necrosis factor-alpha (TNF-α) production [52]. Hence, our findings point to a role of MCFAs in protection against collateral damage caused by free radicals and TNF, which are essential elements of protective immunity against *M. tuberculosis*.

### Complement System Peptide

The peptide C3f is a breakdown product of the C3 component of the complement system, which is generated by the combined actions of factors I and H [53]. C3, a central molecule of the complement cascade, triggers a variety of important immunologic sequellae [54]. Elevated levels of C3f have been correlated with coronary disease and vascular events in women [55]. In our study, the peptide C3f was elevated in female members of the TB^active^ group.

### Uremic Toxins

Significantly reduced serum concentrations in the TB^active^ group of CMPF, 3-indoxyl sulfate (IS), hippurate and indole acetate, suggest uremic cytotoxic activity. Although these compounds have not been analyzed in the context of TB, uremic toxins have been related to vitamin D metabolism [56]. Vitamin D is involved in macrophage activation and has been described as critical for host defense against TB [57]. Reduced serum concentrations in TB patients could be explained by accumulation of respective metabolites in granulomas or by the assumption that vitamin D deficiency is directly related to elevated susceptibility in TB [58], [59]. Moreover, uremic toxins can modulate apoptosis and promote neutrophil clearance [60].

### Stachydrine

Stachydrine and putative demethylated stachydrine (X-11513) were correlated in serum abundance. Their average abundance was only insignificantly elevated in the TB^active^ group. In-depth analysis of the data revealed marked differences amongst participants in this group with eight outliers showing highly elevated serum concentrations of these compounds, compared with only three outliers in the TST^+^ group and four outliers in the TST^–^ group.

Stachydrine and homostachydrine are plant alkaloids found, for example, in alfalfa (lucern, *Medicago sativa*). Stachydrine is also found in *Capparis tomentosa* (Woolly caper-bush), which is used as a traditional remedy in South Africa [61] for treatment of cough and chest pain. These findings emphasize the broad scope of metabolome analyses, which not only encompasses pathogen, host and microbiome metabolism, but also environmental factors such as drug intake.

### Cytokines Linked to Metabolic Changes

The serum levels of several cytokines that were significantly upregulated in samples from TB patients were significantly anticorrelated with the relative serum concentrations of a number of amino acids (see Figure 4). The relative abundance of these amino acids was significantly reduced in the TB^active^ group compared with TST^+^ and TST^–^ groups, whereas cytokines were found at elevated concentrations. However, also within the TB^active^ group these two classes of molecules were negatively correlated. This indicates that in patients with the strongest inflammation, amino acid abundance was diminished and IL-6 and other proinflammatory cytokines were elevated (see Figure 4).

Strongest negative correlations between amino acids and cytokines were observed for the amino acid glutamine. Glutamine is the most abundant amino acid in the body, but can become limiting under stress conditions [62], and consequently impair immune functions [63]. It is tempting to speculate that the observed increase in cytokine serum concentrations accompanied by decreased serum abundance of amino acids are indicators for disease progression in which the organism is gradually depleted of resources, resulting in cachexia. Indeed, the ratio glutamine/glutamate indicates a risk for loss of mass [64]. We found that the glutamine/glutamate ratio in serum of TB^active^ was significantly lower than in TST^–^ and TST^+^ groups (p<2×10^–5^).

The concentrations of G-CSF were significantly anticorrelated with the relative abundances of gamma-glutamyl glutamine and gamma-glutamyl leucine (p<10^–3^, r < –0.55 in the TB^active^ group). This could indicate an effect on gamma-glutamyl transpeptidase, known to be elevated in patients treated with G-CSF [65], [66].

### Specificity of the Biosignature

Since the experimental groups tested did not include patients with diseases other than TB, we cannot ascertain the specificity of the resulting biosignature. It can be expected that several of the observed changes are the result of a general inflammatory process rather than a specific response to TB. Nonetheless, it is tempting to speculate that three clusters of compounds included a specific profile. Both the inosine cluster and fibrinopeptide cluster revealed significant differences between the two healthy groups (TST^–^ and TST^+^), indicating that these effects are specific. Furthermore, we demonstrated that *M. tuberculosis* directly stimulates kynurenine production at the site of infection. We assume that this effect includes a specific pattern because it represents a direct response to the pathogen, although it has been described in other diseases, as well. In addition, TB is linked to general wasting (cachexia), which has been generally observed in TB and AIDS and less so in other infectious diseases. Therefore, there is reason to speculate that definition of a specific metabolite biosignature of TB is feasible.

### Concluding Remarks

TB is a chronic disease known to cause profound metabolic changes [67] as indicated by its various designations as wasting disease, consumption or phthisis in pre-antibiotic times. Metabolomics not only allows robust differentiation of patients with active TB from healthy individuals (be they *M. tuberculosis*-infected or not), it also reveals remarkable differences in the metabolite profile between individual TB cases (see Figure 1A). In contrast, healthy individuals from both TST^–^ and TST^+^ groups were more homogeneous for the most discriminative metabolites.

Our study aimed at a better understanding of the biological processes operative during infection and disease in TB, and we identified several biological mechanisms that apparently play a major role. In addition, our study provides a first step towards the development of metabolomic-based diagnosis of TB. Moreover, understanding host metabolic processes during TB could ultimately inform individualized adjunctive therapy in combination with chemotherapy, notably, during prolonged treatment of patients with multidrug-resistant or extensively drug-resistant TB. Clearly, further development of a tailored biosignature suitable for TB diagnosis will require verification in independent cohorts and comparison with other pulmonary infectious diseases. Yet, our current analyses of several hundred metabolites has led to the definition of a signature comprising fewer than 20 metabolites for reliable discrimination between active TB and latently infected or uninfected healthy individuals, emphasizing the power of metabolomic profiling in TB. It is tempting to speculate that one day, metabolic signatures may be harnessed for the development of a simple and robust dipstick test for point-of-care diagnosis of TB.

## Methods

### Ethics Statement

Human serum samples: the study was approved by the ethical committee of the University of Stellenbosch (Stellenbosch, South Africa) and written informed consent was obtained from all study participants. Human cell samples were obtained in accordance with the local ethical committee (Charite Ethikkommission, Berlin, Germany, EA1/200/08). Animal experiments were performed at the Max Planck Institute for Infection Biology in Berlin, Germany, and experimental protocols were approved by the State Office of Health and Social Affairs (Landesamt für Gesundheit und Soziales), Berlin, Germany.

### Specimens

Serum samples collected at the University of Stellenbosch, in Stellenbosch, South Africa comprised three groups: 46 healthy controls with no clinical signs of TB infection (TST^–^), 46 latently infected subjects with no clinical signs of TB, confirmed by TST (TST^+^) and 44 patients with clinical signs of pulmonary TB (TB^active^; see Table S1). TB patients had the following symptoms: cough for more than 2 weeks and at least two additional symptoms from the following: hemoptysis (coughing up blood), breathing difficulty, fever, night sweats, weight loss, chest pain, fatigue. All patients had two positive (≥1+) sputum smear stains for acid-fast bacilli (Auramine stain or Ziehl-Neelsen). In 24 out of the 34 patients a Mycobacteria Growth Indicator Tube (MGIT) culture was performed, was positive and was speciated for *M. tuberculosis*. All subjects were HIV^–^ and bacille Calmette–Guérin (BCG)-vaccinated as infants, in accordance with the national vaccination program. Subjects were balanced between the groups with respect to age and gender. Clinically relevant information on patients is available upon request. Samples were stored at –80°C. At the time of sample collection, none of the TB patients was a recipient of TB treatment.

### Sample Preparation

Samples were stored at –70°C until processed. Sample preparation was carried out as described previously [68] at Metabolon, Inc. Briefly, recovery standards were added prior to the first step in the extraction process for quality control purposes. To remove protein, dissociate small molecules bound to protein or trapped in the precipitated protein matrix, and to recover chemically diverse metabolites, proteins were precipitated with methanol under vigorous shaking for 2 min (Glen Mills Genogrinder 2000) followed by centrifugation. The resulting extract was divided into four fractions: one for analysis by ultra high performance liquid chromatography-tandem mass spectrometry (UPLC-MS/MS; positive mode), one for analysis by UPLC-MS/MS (negative mode), one for analysis by gas chromatography–mass spectrometry (GC-MS), and one sample was reserved for backup.

Three types of controls were analyzed in concert with the experimental samples: samples generated from a pool of human plasma (extensively characterized by Metabolon, Inc.) served as technical replicate throughout the data set; extracted water samples served as process blanks; and a cocktail of standards spiked into every analyzed sample allowed instrument performance monitoring. Instrument variability was determined by calculating the median relative standard deviation (RSD) for the standards that were added to each sample prior to injection into the mass spectrometers (median RSD = 10%; n = 30 standards). Overall process variability was determined by calculating the median RSD for all endogenous metabolites (i.e., non-instrument standards) present in 100% of the pooled human plasma samples (median RSD = 15%; n = 239 metabolites). Experimental samples and controls were randomized across the platform run.

### Mass Spectrometry Analysis

Non-targeted MS analysis was performed at Metabolon, Inc. Extracts were subjected to either GC-MS [68] or UPLC-MS/MS [69]. The chromatography was standardized and once the method was validated, no further changes were made. As part of Metabolon’s general practice, all columns were purchased from a single manufacturer’s lot at the outset of experiments. All solvents were similarly purchased in bulk from a single manufacturer’s lot in sufficient quantity to complete all related experiments. For each sample, vacuum-dried samples were dissolved in injection solvent containing eight or more injection standards at fixed concentrations, depending on the platform. The internal standards were used both to assure injection and chromatographic consistency. Instruments were tuned and calibrated for mass resolution and mass accuracy daily.

The UPLC-MS/MS platform utilized a Waters Acquity UPLC and a ThermoFisher LTQ mass spectrometer, which included an electrospray ionization source and a linear ion-trap mass analyzer. The instrumentation was set to monitor for positive ions in acidic extracts or negative ions in basic extracts through independent injections. The instrument was set to scan 99–1000 m/z and alternated between MS and MS/MS scans. The scan speed was approximately six scans per s (three MS and three MS/MS scans). MS/MS scans were collected using dynamic exclusion, a process in which after an MS/MS scan of a specific m/z has been obtained, then that m/z is placed on a temporary MS/MS exclude list for a user-set period of time to allow greater MS/MS coverage of ions present in the MS scan because the instrument will not trigger an MS/MS scan of the same ion repeatedly. Extracts were loaded onto columns (Waters UPLC BEH C18-2.1×100 mm, 1.7 µm) and gradient-eluted with water and 95% methanol containing 0.1% formic acid (acidic extracts) or 6.5 mM ammonium bicarbonate (basic extracts). Columns were washed and reconditioned after every injection.

The samples destined for analysis by GC-MS were dried under vacuum desiccation for a minimum of 18 h prior to being derivatized under dried nitrogen using bistrimethyl-silyltrifluoroacetamide. Derivatized samples were separated on a 5% phenyldimethyl silicone column with helium as carrier gas and a temperature ramp from 60° to 340°C within a 17-min period. All samples were analyzed on a Thermo-Finnigan Trace DSQ MS operated at unit mass resolving power with electron impact ionization and a 50–750 atomic mass unit scan range.

### Compound Identification, Quantification, and Data Curation

Metabolites were identified by automated comparison of the ion features in the experimental samples to a reference library of chemical standard entries that included retention time, molecular weight (m/z), preferred adducts, and in-source fragments as well as associated MS spectra and curated by visual inspection for quality control using software developed at Metabolon [70]. Identification of known chemical entities is based on comparison to metabolomic library entries of purified standards. Over 2,400 commercially available purified standard compounds have been acquired and registered into LIMS for distribution to both the LC/MS and GC/MS platforms for determination of their detectable characteristics. An additional 5,300 mass spectral entries have been created for structurally unnamed biochemicals, which have been identified by virtue of their recurrent nature (both chromatographic and mass spectral). These compounds have the potential to be identified by future acquisition of a matching purified standard or by classical structural analysis. Peaks were quantified using area under the curve.

### Multiplex Cytokine/chemokine Analysis

Cytokine and chemokine levels in serum were measured using a premixed 42-plex MILLIPLEX MAP Human Cytokine/Chemokine kit (Millipore GmbH, Germany) according to manufacturer’s instructions. A total of 25 µl of serum was analyzed for the presence of the following analytes: epidermal growth factor (EGF), eotaxin, fibroblast growth factor (FGF)-2, Flt3-L, fractalkine, G-CSF, granulocyte macrophage colony-stimulating factor (GM-CSF), GRO, IFN-α2, IFN-γ, IL-1a, IL-1b, IL-1ra, IL-2, IL-3, IL-4, IL-5, IL-6, IL-7, IL-8, IL-9, IL-10, IL-12p40, IL-12p70, IL-13, IL-15, IL-17, interferon-inducible protein (IP)10, monocyte chemotactic protein (MCP)-1, MCP-3, MDC, macrophage inflammatory protein 1-(MIP1)a, MIP1b, platelet-derived growth factor (PDGF)-AA, PDGF-AB/BB, RANTES, sCD40L, sIL2ra, transforming growth factor-alpha (TGF)-α, TNF-α, TNF-β and VEGF. Cytokine and chemokine levels were measured on a Luminex® 100/200TM system (Luminex Corporation, Austin, TX, USA).

### Experimental TB Infection and Histology

Animals (C57BL/6 mice, 8–12 weeks of age) were infected using a Glas-Col inhalation exposure system with *M. tuberculosis* H37Rv (∼250 colony forming units per mouse). The initial challenge dose was verified 24 h p.i. by plating complete lung homogenates onto Middlebrook 7H11 agar plates. For immunohistochemistry, fixed lung sections (2 µm) were de-waxed, rehydrated and subjected to antigen retrieval, blocking and exposed to goat polyclonal IDO antibody (LifeSpan Biosciences), followed by rabbit anti-goat alkaline phosphatase-conjugated secondary antibody. Alkaline phosphatase activity was visualized using new fuchsin (Dako). Sections were counterstained with hemalaun (Merck).

### Ex-vivo Human Cells

Human DCs and macrophages were generated from buffy coats obtained from healthy volunteers recruited at the German Red Cross (Deutsches Rotes Kreuz). Peripheral blood mononuclear cells were recovered from interphase of Ficoll gradient according to standard protocols. To obtain untouched monocytes, cells were subjected to negative MACS selection using the Monocyte Isolation Kit II (Miltenyi Biotec). Flow-through cells were collected and grown in complete RPMI1640 medium containing 800 U/ml hGM-CSF and 500 U/ml hIL-4 (Strathmann) for 7 days to obtain immature DCs. Macrophages were generated from CD14^+^ cells by adherence to plastic culture flasks. For T cell assays, cells from healthy PPD^+^ donors were used to obtain DCs and T cells. DCs were generated as previously described and CD3^+^CD4^+^ and CD3^+^CD8^+^ T cells were obtained by negative MACS selection according to manufacturer’s instructions (Pan T cell Isolation Kit, Miltenyi).

### Kynurenine Detection

Cell culture supernatants from infected and stimulated cells (DCs and macrophages) were deproteinized and kynurenines and tryptophan concentrations were measured by high-performance liquid chromatography using a Waters reverse phase C-18 column with a photodiode array detector (Waters 996). IDO1 enzymatic activity was assessed as a direct function of kynurenine production [71].

### T Cell Assay

Monocyte-derived DCs treated with 1-methyl-tryptophan-DL (1-MT-DL) or with 1-MT-DL plus 3-OH-kynurenine (Kyn) and pulsed with PPD (10 µg/ml) and ManLAM (15 µg/ml) were cocultured with autologous T cells at a ratio of 1∶20 (10^4^ DCs and 2×10^5^ T cells in triplicates). T cells were labeled with carboxyfluorescein diacetate succinimidyl ester (CFSE, CellTrace CFSE Cell Proliferation Kit, Molecular Probes) at 2.5 uM according to manufacturer’s instructions. After 4 days of coculture, T cell proliferation was assessed by flow cytometry using anti-CD3, -CD4 and -CD8 monoclonal antibodies (all from BD) in combination with CFSE. Lymphocytes were gated based on forward and side scatter, and propidium iodide-positive cells were excluded from the analysis. The reduction in CFSE fluorescence intensity was analyzed on CD3^+^CD4^+^ and CD3^+^CD8^+^ gated cells. The ratio of proliferating to nonproliferating T cells was calculated and normalized to untreated controls. Data were analyzed by Anova with Dunnet’s multiple comparison test.

### Quantitative RT-PCR

RNA was isolated with TRIzol (Invitrogen) and High Pure RNA Isolation Kit (Roche). cDNA was synthesized using the SuperScript III First-Strand Synthesis System and random hexamer primers (Invitrogen). RT-PCR was performed in triplicates using the ABIPRISM SDS 7900 system (Applied Biosystems). Fragments were amplified using the SYBR Green I Reaction Mix (Applied Biosystems) and specific primers for IDO1 (Hs_IDO1_1_SG QuantiTect Primer Assay, Qiagen) and normalized to the expression of human acidic ribosomal protein (HuPO) housekeeping gene (F-5′-GCTTCCTGGAGGGTGTCC-3′; R-5′-GGACTCGTTTGTACCCGTTG-3′). Threshold cycle values CT for each PCR product were determined using ABIPRISM SDS7900 software and ΔCT values were calculated. Fold differences (FD) in expression levels between IDO1 and HUPO were calculated according to the formula FD = 2^ΔCT^.

### Statistical Analysis

Relative abundances of biochemical compounds obtained by MS were normalized using the aquantile normalisation method from the R package limma [72] and used as an entry point for further statistical analyses. For a general comparison, biochemical compounds with altered levels in the different groups (TST^–^, TST^+^ and TB^active^) were obtained and compared using t-test and Wilcoxon sum rank test (see Table S2). Significant p values were adjusted for multiple testing using the Benjamini and Hochberg correction [73]. Also, several groups of biochemically similar compounds were tested in an ANOVA. Second, a RF classifier was used to identify biomarkers sufficiently distinct between groups. Functional linkage between different compounds was obtained by first fitting a simple linear model for each compound and subsequently calculating the correlation coefficient between the models’ residuals applying a bootstrapping procedure. In the applied linear model Y = aX+b, where Y equals group classification (TST^–^, TST^+^ and TB^active^) and X is the vector of the relative levels of the compounds. R scripts used to generate the data are available upon request. Significantly correlated compound profiles were used to infer clusters of functionally related small biochemical compounds. To explore the link between metabolic and cytokine profiles, we calculated Spearman correlation coefficients between the analyzed cytokine profiles and small metabolic compounds, and corrected for multiple testing. Next, to determine which categories of metabolic compounds correlated with cytokine and chemokine profiles, we performed an enrichment analysis using a hypergeometric test for the main categories of metabolic compounds (amino acids, carbohydrates, cofactors and vitamins, energy metabolism, lipids, nucleotides, peptides and xenobiotics).

### Classification Analysis

The predictive value of small metabolic compounds as biomarkers in TB diagnosis was estimated using a supervised machine learning algorithm [23], as implemented in the R package “randomForest” (RF) [74]. The run parameters selected for sensitivity were mtry = 50 and ntree = 1,000. The classifiers were obtained for all three experimental groups as one data set, and in pairwise comparisons between each of the two groups as another data set. The findings were independently confirmed using SPLS-DA [24]. To estimate the number of variables needed for a robust classification, we applied an external leave-one-out (LOO) cross-validation. In each LOO round, one sample was removed from the data set. An RF model was constructed based on all variables, and the variables were sorted based on the importance from that model. Next, a series of secondary RF models were constructed based on the top 2, 3, 4, etc. variables from the importance list. Each of these secondary models was then validated against the LOO sample. The results were summarized for each number of variables tested, and plotted on Figure S1.

### Clustering

Biochemical compounds were clustered using normalized relative compound levels and averaged hierarchical clustering, correlation-based distance and with bootstrapping implemented in the R package pvclust [75]. Clusters obtained for 10,000 bootstraps with approximately unbiased (AU) bootstrap confidence values >0.95 were considered significant.

### Data Availability

All data, cluster assignments, scripts, and additional plots are available upon request.

### List of Abbreviations

1-MT-DL, 1-methyl-DL-tryptophan

AU, approximately unbiased

BCG, bacille Calmette-Guérin

CFSE, carboxyfluorescein diacetate succinimidyl ester

CMPF, 3-carboxy-4-methyl-5-propyl-2-furanpropanoic acid

CXCL-10, C-X-C motif chemokine 10

DC, dendritic cell

EGF, epidermal growth factor

FGF, fibroblast growth factor

GC–MS, gas chromatography–mass spectrometry

G-CSF, granulocyte colony-stimulating factor

GITR, glucocorticoid-induced tumor necrosis factor receptor

GM-CSF, granulocyte macrophage colony-stimulating factor

HPLC, high-performance liquid chromatography

HuPO, human acidic ribosomal protein

IDO1, indoleamine 2,3 dioxygenase 1

IFN-γ, interferon-gamma

IL, interleukin

IP, interferon-inducible protein

LC–MSMS, liquid chromatography-tandem mass spectrometry

LOO, leave-one-out cross-validation

LPCs, lysophosphatidylcholines

ManLAM, mannosylated lipoarabinomannan

MCFA, medium-chain fatty acid

MCP, monocyte chemotactic protein

MDC, macrophage-derived cytokine

MGIT, Mycobacteria Growth Indicator Tube

MIP, macrophage inflammatory protein

NAM, N-acetyl-L-methionine

NMR, nuclear magnetic resonance

PDGF, platelet-derived growth factor

PPD, purified protein derivative

RF, random forests

sIL2ra, soluble alpha chain of the IL-2 receptor

SPLS-DA, sparse PLS discriminant analysis

TB, tuberculosis

TB^active^, patients with active TB

TGF, transforming growth factor

TNF, tumor necrosis factor

TST, tuberculin skin test

TST^–^, healthy *M. tuberculosis*-uninfected controls

TST^+^, latently *M. tuberculosis*-infected healthy individuals

VEGF, vascular endothelial growth factor

## Supporting Information

Figure S1 **Fifteen to twenty metabolites suffice to distinguish between TB patients and latently infected groups.** Figure shows the decrease of the average classification error rate as a function of the number of different small metabolic compounds chosen for the classification. Error bars denote standard error of the mean of 50 re-sampling procedures.
(TIF)

Figure S2 **Heatmap showing relative levels of small metabolic compounds in TB patients (TB** ***^active^*** **) and latently infected individuals (TST^+^).** For purposes of illustration, 10 profiles from each group were randomly assigned to a test set, and calculations were repeated for the remaining training set. Left, training-set levels; only 50 compounds selected by variable importance from the RF model were chosen. Right, test-set levels; all test set samples were correctly assigned to a study class.
(TIF)

Figure S3 **Network showing functional relationships between the small metabolic compounds in TB patients, healthy uninfected and latently infected individuals.** Nodes correspond to metabolites; edges correspond to statistically significant correlation between residual small metabolite profiles corrected for study classes. Colors correspond to differences between the TST^–^ and TB*^active^* (A), TST^+^ and TB*^active^* (B) or TST^–^ and TST^+^ classes (C). Color intensity indicates significance of difference with darker colors corresponding to more significant differences. Metabolites with adjusted p value >0.05 are not colored. Line widths correspond to the absolute Spearman correlation coefficients corrected for groups (see “Methods”). Figure S3.A is the same as Figure 3 in the Manuscript and has been included here for completeness.
(TIF)

Table S1 **Demographic characteristics of study subjects.**
(DOCX)

Table S2 **Significant differences between serum concentrations of small metabolic compounds between each two groups.** A), differences between TST^-^ and TST^+^; B), differences between TST^-^ and TB*^active^*; C) differences between TST^+^ and TB*^active^*. ***p*** denotes the p value from Wilcoxon rank sum test; ***q*** is the p value corrected for multiple testing using false discovery rate correction (Benjamini 1995). Only compounds for which the ***q*** value was smaller than 0.01 are shown.
(DOCX)

Table S3 **Top 50 compounds sorted by importance from the random forests analysis of biochemical compound discriminatory power between TST^+^ and TB** ***^active^*** **.** Compounds with names starting with an “X” could not be uniquely identified in the analysis. The columns TST^−^, TST^+^ and TB*^active^* give the average relative abundance levels in the corresponding study groups. Top twenty compounds are sufficient to sensitively discriminate latent infection from active TB. See text for details.
(DOCX)

Table S4 **Significant clusters of small metabolic compounds containing at least five different members.**
(DOCX)
