# Correction: Biomarkers of Inflammation, Immunosuppression and Stress with Active Disease Are Revealed by Metabolomic Profiling of Tuberculosis Patients

**DOI:** 10.1371/annotation/b7f554bc-ad78-4745-9cd6-e14954d6a01d

**Published:** 2012-12-26

**Authors:** January Weiner, Shreemanta K. Parida, Jeroen Maertzdorf, Gillian F. Black, Dirk Repsilber, Anna Telaar, Robert P. Mohney, Cordelia Arndt-Sullivan, Christian A. Ganoza, Kellen C. Faé, Gerhard Walzl, Stefan H. E. Kaufmann

There was an error in the title of this article. The correct title is: Biomarkers of Inflammation, Immunosuppression and Stress Are Revealed by Metabolomic Profiling of Tuberculosis Patients.

The correct citation is: Weiner J 3rd, Parida SK, Maertzdorf J, Black GF, Repsilber D, et al. (2012) Biomarkers of Inflammation, Immunosuppression and Stress Are Revealed by Metabolomic Profiling of Tuberculosis Patients. PLoS ONE 7(7): e40221. doi:10.1371/journal.pone.0040221

