# Correction: Biomarkers of Inflammation, Immunosuppression and Stress Are Revealed by Metabolomic Profiling of Tuberculosis Patients

**DOI:** 10.1371/journal.pone.0153050

**Published:** 2016-03-31

**Authors:** The PLOS ONE Staff

## Notice of Republication

The original Supporting Information files were published in error. This article was republished on March 23, 2016, and the files removed from the HTML and PDF versions. The publisher apologizes for the error.

Additionally, this republication addresses the error in the title that was corrected on December 26, 2012 [2].

Please download this article again to view the correct version.
