# Supplementary figures and images for: Biomarkers of Inflammation, Immunosuppression and Stress Are Revealed by Metabolomic Profiling of Tuberculosis Patients
Source: PLoS One. 2012 Jul 23;7(7):e40221. doi: 10.1371/journal.pone.0040221 (PMC3402490; doi:10.1371/journal.pone.0040221)

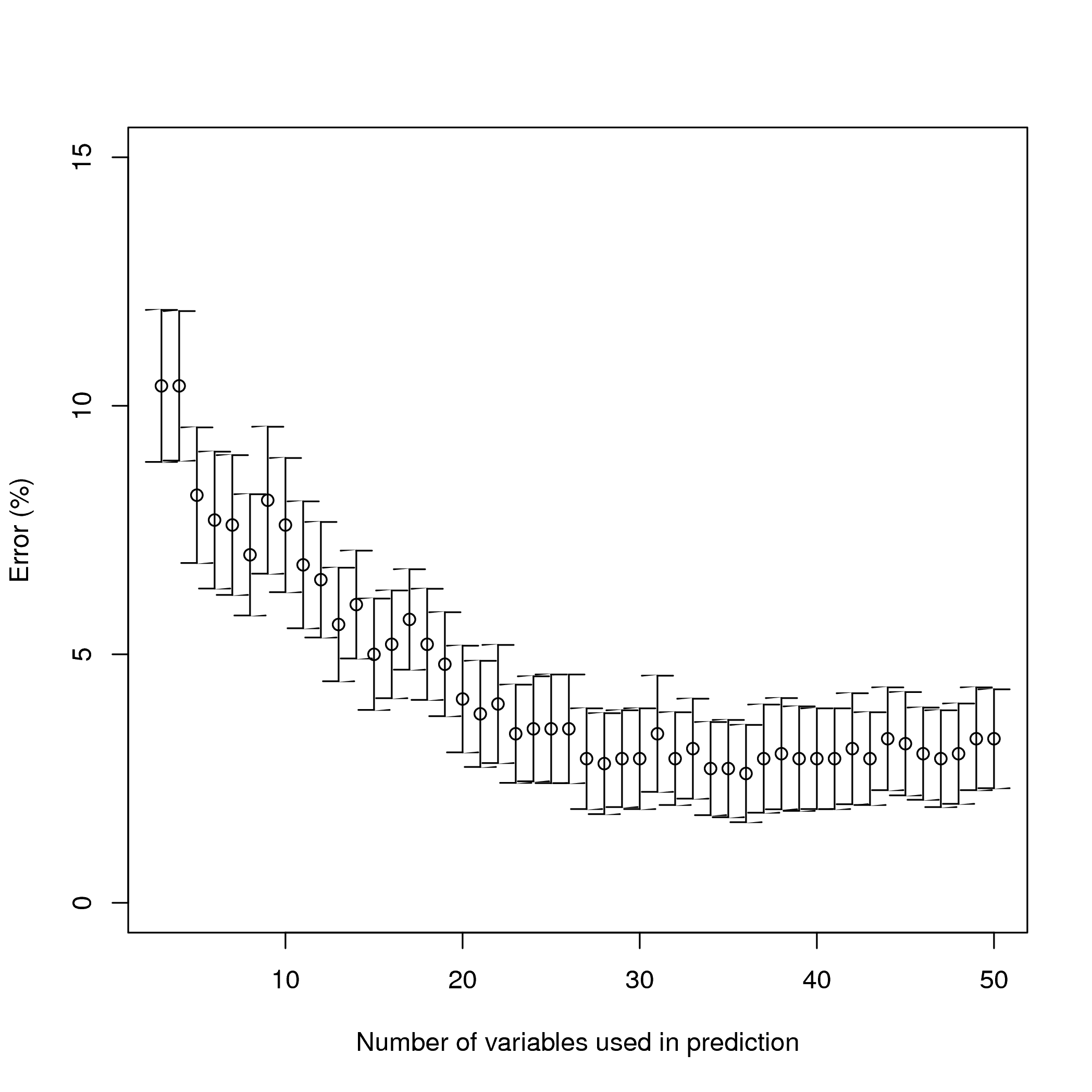

Supplement: Figure S1 — Fifteen to twenty metabolites suffice to distinguish between TB patients and latently infected groups. Figure shows the decrease of the average classification error rate as a function of the number of different small metabolic compounds chosen for the classification. Error bars denote standard error of the mean of 50 re-sampling procedures. (TIF) [file pone.0040221.s001.tif]

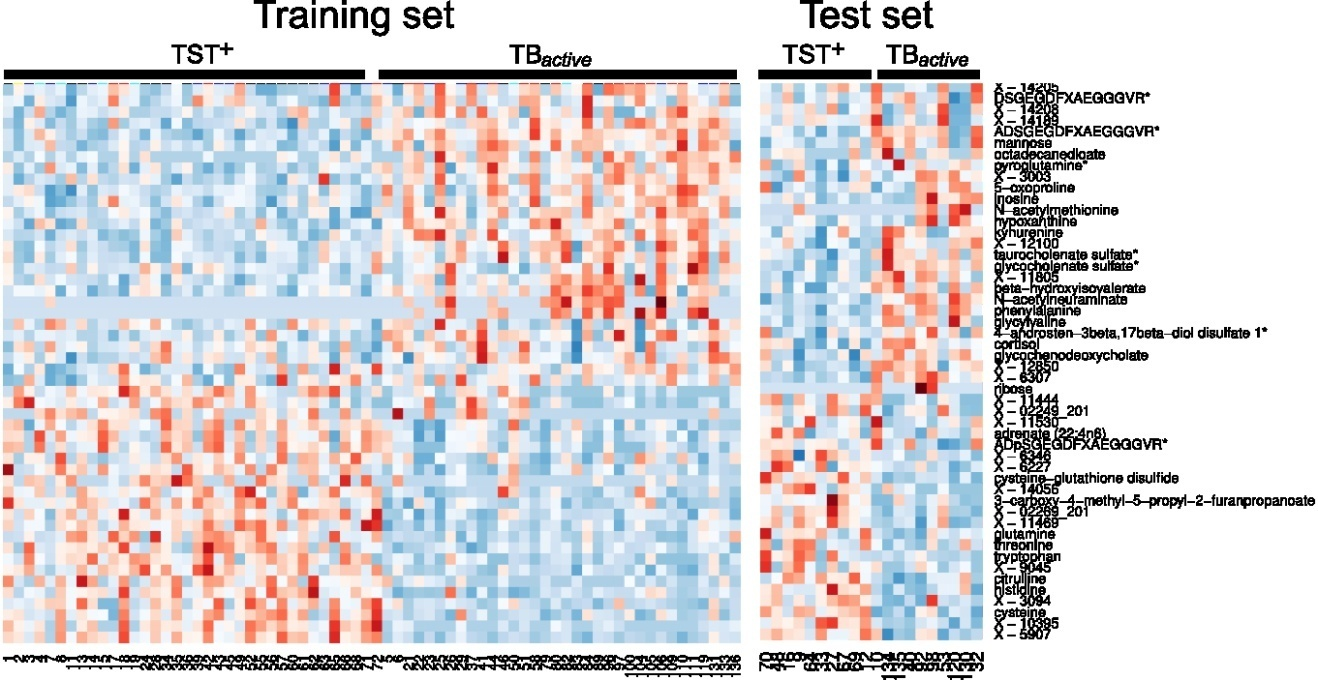

Supplement: Figure S2 — Heatmap showing relative levels of small metabolic compounds in TB patients (TB active ) and latently infected individuals (TST+). For purposes of illustration, 10 profiles from each group were randomly assigned to a test set, and calculations were repeated for the remaining training set. Left, training-set levels; only 50 compounds selected by variable importance from the RF model were chosen. Right, test-set levels; all test set samples were correctly assigned to a study class. (TIF) [file pone.0040221.s002.tif]

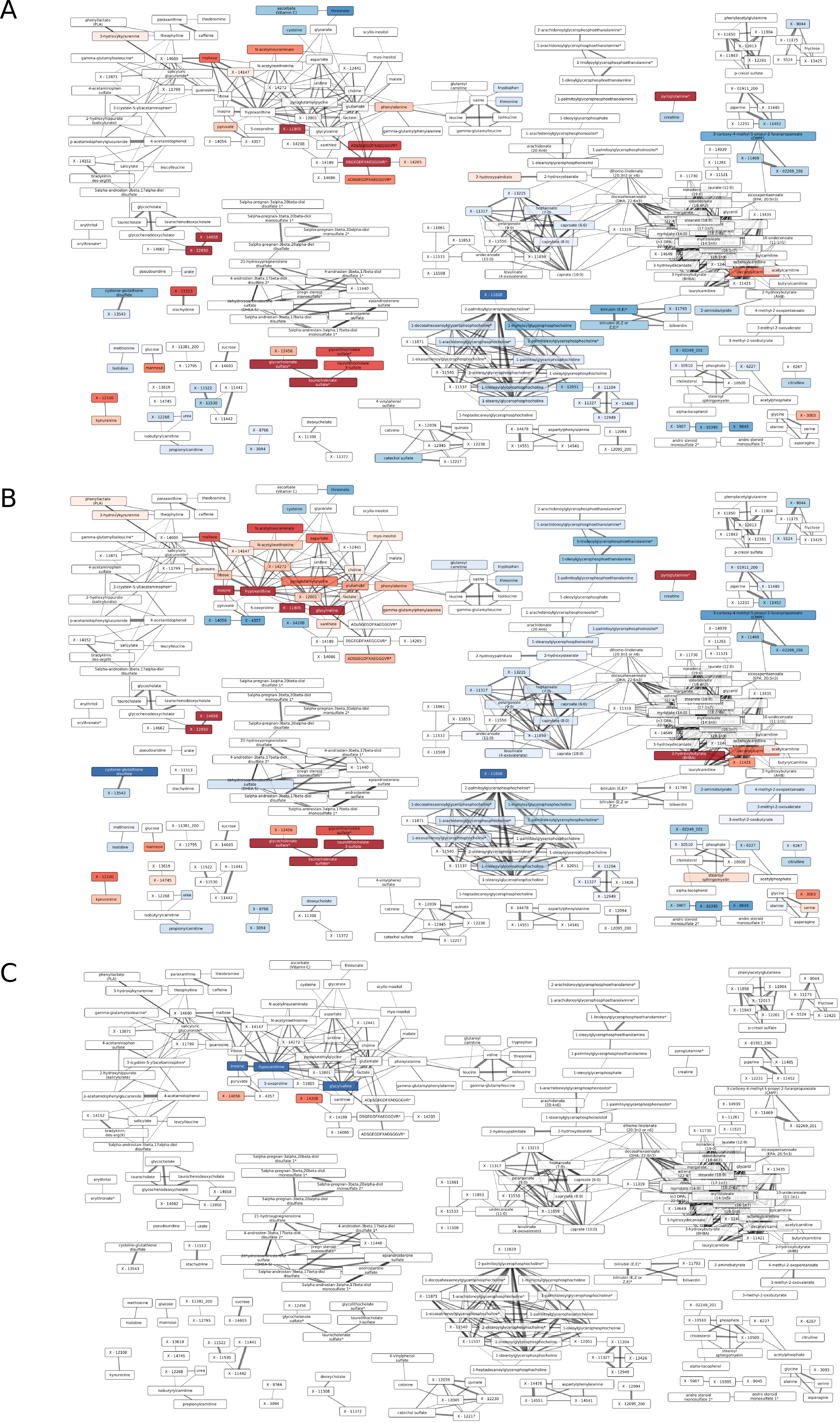

Supplement: Figure S3 — Network showing functional relationships between the small metabolic compounds in TB patients, healthy uninfected and latently infected individuals. Nodes correspond to metabolites; edges correspond to statistically significant correlation between residual small metabolite profiles corrected for study classes. Colors correspond to differences between the TST– and TBactive (A), TST+ and TBactive (B) or TST– and TST+ classes (C). Color intensity indicates significance of difference with darker colors corresponding to more significant differences. Metabolites with adjusted p value >0.05 are not colored. Line widths correspond to the absolute Spearman correlation coefficients corrected for groups (see “Methods”). Figure S3.A is the same as Figure 3 in the Manuscript and has been included here for completeness. (TIF) [file pone.0040221.s003.tif]
