# Supplementary material for: Biomarkers of Inflammation, Immunosuppression and Stress Are Revealed by Metabolomic Profiling of Tuberculosis Patients
Source: PLoS One. 2012 Jul 23;7(7):e40221. doi: 10.1371/journal.pone.0040221 (PMC3402490; doi:10.1371/journal.pone.0040221)
Supplement: Table S1 — Demographic characteristics of study subjects. (DOCX) [file pone.0040221.s004.docx]

**Table S1.** Demographic characteristics of study subjects.

| **Group** | **Description** | **n** | **Age range, years (SD)** | **Gender ratio (M/F)** |
| --- | --- | --- | --- | --- |
| TST^–^ | Negative controls without *M. tuberculosis* infection | 46 | 10–62 (27.7 ± 15.0) | 29/17 |
| TST^+^ | Latent *M. tuberculosis*-infected individuals | 46 | 12–57 (27.4 ± 11.9) | 29/17 |
| TB*^active^* | Patients with active TB symptoms | 44 | 14–49 (26 ± 9.2) | 25/19 |

Tablenotes: Active TB disease, TB*^active^*; female, F; male, M; *Mycobacterium tuberculosis*, *M. tuberculosis*; number, n; standard deviation, SD; tuberculin skin test (TST) negative, TST^–^; TST positive, TST^+^.
