# Supplementary material for: Biomarkers of Inflammation, Immunosuppression and Stress Are Revealed by Metabolomic Profiling of Tuberculosis Patients
Source: PLoS One. 2012 Jul 23;7(7):e40221. doi: 10.1371/journal.pone.0040221 (PMC3402490; doi:10.1371/journal.pone.0040221)
Supplement: Table S2 — Significant differences between serum concentrations of small metabolic compounds between each two groups. A), differences between TST- and TST+; B), differences between TST- and TBactive; C) differences between TST+ and TBactive. p denotes the p value from Wilcoxon rank sum test; q is the p value corrected for multiple testing using false discovery rate correction (Benjamini 1995). Only compounds for which the q value was smaller than 0.01 are shown. (DOCX) [file pone.0040221.s005.docx]

**Table S2.** Significant differences between serum concentrations of small metabolic compounds between each two groups. A), differences between TST^-^ and TST^+^; B), differences between TST^-^ and TB*^active^*; C) differences between TST^+^ and TB*^active^*. ***p*** denotes the p value from Wilcoxon rank sum test; ***q*** is the p value corrected for multiple testing using false discovery rate correction (Benjamini 1995). Only compounds for which the ***q*** value was smaller than 0.01 are shown

A)

| **Name** | **p** | **q** |
| --- | --- | --- |
| DSGEGDFXAEGGGVR* | 1.24E-06 | 0.00017 |
| ribose | 6.61E-07 | 0.00017 |
| X - 14208 | 9.63E-07 | 0.00017 |
| ADpSGEGDFXAEGGGVR* | 2.49E-06 | 0.00026 |
| glycylvaline | 4.70E-06 | 0.00040 |
| hypoxanthine | 9.32E-06 | 0.00066 |
| 5-oxoproline | 1.87E-05 | 0.00113 |
| X - 14205 | 4.53E-05 | 0.00239 |
| N-acetylmethionine | 1.14E-04 | 0.00537 |
| X - 14189 | 1.74E-04 | 0.00735 |

B)

| **name** | **p** | **q** |
| --- | --- | --- |
| X - 10395 | 6.43E-014 | 2.73E-011 |
| X - 12100 | 4.72E-011 | 1.00E-008 |
| X - 9045 | 3.80E-010 | 5.37E-008 |
| histidine | 2.30E-009 | 2.44E-007 |
| citrulline | 4.07E-009 | 3.13E-007 |
| X - 6307 | 4.43E-009 | 3.13E-007 |
| cysteine | 1.18E-008 | 7.12E-007 |
| glycocholenate sulfate* | 2.22E-008 | 1.17E-006 |
| X - 02249_201 | 3.06E-008 | 1.44E-006 |
| tryptophan | 3.57E-008 | 1.52E-006 |
| mannose | 5.93E-008 | 2.29E-006 |
| ADSGEGDFXAEGGGVR* | 9.50E-008 | 3.36E-006 |
| glutamine | 2.62E-007 | 8.55E-006 |
| taurocholenate sulfate* | 4.14E-007 | 1.26E-005 |
| X - 12850 | 5.56E-007 | 1.57E-005 |
| pyroglutamine* | 7.97E-007 | 1.99E-005 |
| X - 6346 | 7.63E-007 | 1.99E-005 |
| X - 11444 | 9.01E-007 | 2.12E-005 |
| DSGEGDFXAEGGGVR* | 1.30E-006 | 2.91E-005 |
| HWESASXX* | 1.66E-006 | 3.51E-005 |
| X - 5907 | 1.90E-006 | 3.83E-005 |
| X - 14658 | 2.35E-006 | 4.52E-005 |
| citrate | 2.83E-006 | 5.22E-005 |
| X - 3094 | 3.25E-006 | 5.74E-005 |
| cortisol | 3.45E-006 | 5.85E-005 |
| N-acetylneuraminate | 3.96E-006 | 6.45E-005 |
| creatine | 6.17E-006 | 9.69E-005 |
| 1-myristoylglycerophosphocholine | 9.96E-006 | 0.00015 |
| X - 07765_201 | 1.12E-005 | 0.00016 |
| X - 09789_201 | 1.40E-005 | 0.00020 |
| threonine | 1.48E-005 | 0.00020 |
| kynurenine | 1.91E-005 | 0.00025 |
| X - 11805 | 1.88E-005 | 0.00025 |
| phenylalanine | 1.98E-005 | 0.00025 |
| taurochenodeoxycholate | 2.24E-005 | 0.00027 |
| beta-hydroxyisovalerate | 2.39E-005 | 0.00028 |
| 2-aminobutyrate | 3.90E-005 | 0.00043 |
| 3-hydroxykynurenine | 3.89E-005 | 0.00043 |
| X - 13543 | 3.97E-005 | 0.00043 |
| octadecanedioate | 4.12E-005 | 0.00044 |
| X - 12844 | 4.48E-005 | 0.00046 |
| X - 9044 | 5.20E-005 | 0.00053 |
| threonate | 6.12E-005 | 0.00059 |
| X - 11204 | 6.09E-005 | 0.00059 |
| trans-4-hydroxyproline | 7.51E-005 | 0.00069 |
| X - 11530 | 7.48E-005 | 0.00069 |
| 1-linoleoylglycerophosphocholine | 9.18E-005 | 0.00081 |
| X - 02269_201 | 9.10E-005 | 0.00081 |
| glycochenodeoxycholate | 0.00012 | 0.00102 |

B) (continued)

| **Name** | **p** | **q** |
| --- | --- | --- |
| cysteine-glutathione disulfide | 0.00013 | 0.00106 |
| glycolithocholate sulfate* | 0.00013 | 0.00106 |
| X - 12851 | 0.00013 | 0.00106 |
| X - 6227 | 0.00013 | 0.00106 |
| 3-carboxy-4-methyl-5-propyl-2-furanpropanoate (CMPF) | 0.00014 | 0.00107 |
| 1-palmitoleoylglycerophosphocholine* | 0.00019 | 0.00146 |
| alpha-hydroxyisovalerate | 0.00020 | 0.00150 |
| X - 11549 | 0.00020 | 0.00152 |
| X - 11452 | 0.00030 | 0.00221 |
| X - 11538 | 0.00031 | 0.00225 |
| betaine | 0.00042 | 0.00295 |
| 4-vinylphenol sulfate | 0.00043 | 0.00301 |
| X - 12949 | 0.00045 | 0.00309 |
| X - 11820 | 0.00061 | 0.00410 |
| catechol sulfate | 0.00064 | 0.00423 |
| X - 11317 | 0.00071 | 0.00454 |
| X - 12734 | 0.00070 | 0.00454 |
| X - 12051 | 0.00072 | 0.00454 |
| X - 11793 | 0.00073 | 0.00455 |
| 1-palmitoylglycerophosphocholine | 0.00076 | 0.00468 |
| X - 11327 | 0.00078 | 0.00475 |
| taurolithocholate 3-sulfate | 0.00089 | 0.00533 |
| isovalerate | 0.00097 | 0.00551 |
| piperine | 0.00098 | 0.00551 |
| X - 12510 | 0.00099 | 0.00551 |
| X - 13425 | 0.00097 | 0.00551 |
| X - 14662 | 0.00099 | 0.00551 |
| X - 12456 | 0.00104 | 0.00570 |
| X - 3003 | 0.00106 | 0.00577 |
| indoleacetate | 0.00116 | 0.00624 |
| 3-phenylpropionate (hydrocinnamate) | 0.00120 | 0.00630 |
| X - 12268 | 0.00120 | 0.00630 |
| bilirubin (E,E)* | 0.00130 | 0.00670 |
| stachydrine | 0.00134 | 0.00684 |
| methionine | 0.00145 | 0.00730 |
| ADpSGEGDFXAEGGGVR* | 0.00153 | 0.00756 |
| ascorbate (Vitamin C) | 0.00157 | 0.00756 |
| indolepropionate | 0.00157 | 0.00756 |
| X - 11522 | 0.00156 | 0.00756 |
| X - 12536 | 0.00165 | 0.00788 |
| X - 11470 | 0.00189 | 0.00892 |
| taurocholate | 0.00214 | 0.00998 |

C)

| **Name** | **p** | **q** |
| --- | --- | --- |
| X - 10395 | 4.04E-14 | 1.71E-11 |
| X - 12100 | 3.42E-11 | 7.24E-09 |
| histidine | 9.23E-11 | 1.30E-08 |
| cysteine | 2.67E-10 | 2.26E-08 |
| cysteine-glutathione disulfide | 2.33E-10 | 2.26E-08 |
| X - 3094 | 4.60E-10 | 3.25E-08 |
| citrulline | 3.04E-09 | 1.84E-07 |
| N-acetylneuraminate | 4.07E-09 | 1.97E-07 |
| X - 3003 | 4.17E-09 | 1.97E-07 |
| X - 9045 | 9.21E-09 | 3.91E-07 |
| tryptophan | 1.83E-08 | 7.05E-07 |
| X - 13543 | 4.62E-08 | 1.63E-06 |
| glycocholenate sulfate* | 6.63E-08 | 1.76E-06 |
| phenylalanine | 6.63E-08 | 1.76E-06 |
| X - 14056 | 5.88E-08 | 1.76E-06 |
| X - 5907 | 5.77E-08 | 1.76E-06 |
| glutamine | 7.40E-08 | 1.84E-06 |
| 3-carboxy-4-methyl-5-propyl-2-furanpropanoate (CMPF) | 8.51E-08 | 1.90E-06 |
| X - 6307 | 8.47E-08 | 1.90E-06 |
| X - 11805 | 1.80E-07 | 3.81E-06 |
| X - 02269_201 | 2.51E-07 | 5.07E-06 |
| cortisol | 3.51E-07 | 6.77E-06 |
| trans-4-hydroxyproline | 7.48E-07 | 1.38E-05 |
| gamma-glutamylglutamine | 8.99E-07 | 1.53E-05 |
| pyroglutamine* | 8.83E-07 | 1.53E-05 |
| X - 6227 | 1.17E-06 | 1.85E-05 |
| X - 8766 | 1.18E-06 | 1.85E-05 |
| X - 6346 | 1.59E-06 | 2.41E-05 |
| kynurenine | 1.76E-06 | 2.42E-05 |
| ribose | 1.77E-06 | 2.42E-05 |
| X - 11469 | 1.73E-06 | 2.42E-05 |
| mannose | 2.43E-06 | 3.21E-05 |
| X - 02249_201 | 2.84E-06 | 3.65E-05 |
| 1-linoleoylglycerophosphoethanolamine* | 2.95E-06 | 3.68E-05 |
| caprylate (8:0) | 4.18E-06 | 5.06E-05 |
| threonine | 4.45E-06 | 5.10E-05 |
| X - 13215 | 4.33E-06 | 5.10E-05 |
| N-acetylmethionine | 6.12E-06 | 6.83E-05 |
| X - 9044 | 6.59E-06 | 7.17E-05 |
| taurocholenate sulfate* | 6.92E-06 | 7.34E-05 |
| octadecanedioate | 9.89E-06 | 1.02E-04 |
| X - 12456 | 1.02E-05 | 1.03E-04 |
| 1-linoleoylglycerophosphocholine | 1.08E-05 | 1.07E-04 |
| glycylvaline | 1.25E-05 | 1.21E-04 |
| citrate | 1.35E-05 | 1.22E-04 |
| X - 12850 | 1.32E-05 | 1.22E-04 |
| X - 14658 | 1.33E-05 | 1.22E-04 |
| hypoxanthine | 1.40E-05 | 1.24E-04 |
| creatine | 1.75E-05 | 1.51E-04 |

C) (continued)

| **Name** | **p** | **q** |
| --- | --- | --- |
| X - 14208 | 1.79E-05 | 1.52E-04 |
| C-glycosyltryptophan* | 3.04E-05 | 2.53E-04 |
| pyroglutamylglycine | 3.13E-05 | 2.55E-04 |
| inosine | 3.63E-05 | 2.85E-04 |
| X - 09789_201 | 3.60E-05 | 2.85E-04 |
| 3-hydroxykynurenine | 3.89E-05 | 3.00E-04 |
| pelargonate (9:0) | 4.80E-05 | 3.64E-04 |
| heptanoate (7:0) | 5.71E-05 | 4.25E-04 |
| urea | 6.54E-05 | 4.78E-04 |
| X - 11820 | 7.16E-05 | 5.14E-04 |
| 2-aminobutyrate | 7.76E-05 | 5.49E-04 |
| glutamate | 7.89E-05 | 5.49E-04 |
| taurochenodeoxycholate | 8.24E-05 | 5.63E-04 |
| threonate | 9.99E-05 | 6.72E-04 |
| X - 9108 | 1.03E-04 | 6.82E-04 |
| bilirubin (Z,Z) | 1.19E-04 | 7.68E-04 |
| X - 11317 | 1.20E-04 | 7.68E-04 |
| X - 11859 | 1.22E-04 | 7.70E-04 |
| caproate (6:0) | 1.26E-04 | 7.83E-04 |
| beta-hydroxyisovalerate | 2.31E-04 | 0.0014 |
| X - 11444 | 2.66E-04 | 0.0016 |
| alpha-hydroxyisovalerate | 2.72E-04 | 0.0016 |
| X - 07765_201 | 2.80E-04 | 0.0016 |
| X - 12844 | 2.85E-04 | 0.0017 |
| X - 14147 | 3.31E-04 | 0.0019 |
| xanthine | 4.51E-04 | 0.0025 |
| X - 13425 | 4.46E-04 | 0.0025 |
| isovalerate | 4.62E-04 | 0.0025 |
| allantoin | 4.81E-04 | 0.0026 |
| inositol 1-phosphate (I1P) | 4.82E-04 | 0.0026 |
| 3-methyl-2-oxovalerate | 5.18E-04 | 0.0027 |
| ADSGEGDFXAEGGGVR* | 6.29E-04 | 0.0033 |
| X - 4357 | 6.68E-04 | 0.0035 |
| 4-methyl-2-oxopentanoate | 7.29E-04 | 0.0037 |
| 3-phenylpropionate (hydrocinnamate) | 7.82E-04 | 0.0039 |
| X - 11452 | 8.18E-04 | 0.0041 |
| 1-myristoylglycerophosphocholine | 8.38E-04 | 0.0041 |
| catechol sulfate | 9.07E-04 | 0.0044 |
| methionine | 9.06E-04 | 0.0044 |
| taurolithocholate 3-sulfate | 9.45E-04 | 0.0045 |
| maltose | 9.99E-04 | 0.0047 |
| indolelactate | 0.0011 | 0.0050 |
| X - 11538 | 0.0011 | 0.0050 |
| X - 14189 | 0.0011 | 0.0051 |
| proline | 0.0013 | 0.0057 |
| X - 14588 | 0.0018 | 0.0078 |
| 2-hydroxystearate | 0.0018 | 0.0080 |
| 1-oleoylglycerophosphoethanolamine | 0.0019 | 0.0081 |
| X - 11204 | 0.0019 | 0.0081 |
| X - 12851 | 0.0020 | 0.0084 |
| propionylcarnitine | 0.0021 | 0.0088 |
| serine | 0.0022 | 0.0092 |
| 1-stearoylglycerophosphoinositol | 0.0022 | 0.0092 |
| phosphate | 0.0022 | 0.0092 |
| glycolithocholate sulfate* | 0.0024 | 0.0099 |
