# Supplementary material for: Biomarkers of Inflammation, Immunosuppression and Stress Are Revealed by Metabolomic Profiling of Tuberculosis Patients
Source: PLoS One. 2012 Jul 23;7(7):e40221. doi: 10.1371/journal.pone.0040221 (PMC3402490; doi:10.1371/journal.pone.0040221)
Supplement: Table S3 — Top 50 compounds sorted by importance from the random forests analysis of biochemical compound discriminatory power between TST+ and TB active . Compounds with names starting with an “X” could not be uniquely identified in the analysis. The columns TST−, TST+ and TBactive give the average relative abundance levels in the corresponding study groups. Top twenty compounds are sufficient to sensitively discriminate latent infection from active TB. See text for details. (DOCX) [file pone.0040221.s006.docx]

**Table S3.** Top 50 compounds sorted by importance from the random forests analysis of biochemical compound discriminatory power between TST^+^ and TB*^active^*. Compounds with names starting with an “X” could not be uniquely identified in the analysis. The columns TST^‒^, TST^+^ and TB*^active^* give the average relative abundance levels in the corresponding study groups. Top twenty compounds are sufficient to sensitively discriminate latent infection from active TB. See text for details.

| **BIOCHEMICAL NAME** | **Pathway** | **KEGG ID** | **TST^–^** | **TST^+^** | **TB*^active^*** |
| --- | --- | --- | --- | --- | --- |
| X - 10395 | No Pathway |  | 1.18 | 1.33 | 0.48 |
| X - 3094 | No Pathway |  | 1.17 | 1.24 | 0.85 |
| X - 12100 | No Pathway |  | 0.95 | 0.91 | 1.72 |
| Histidine | Histidine metabolism | C00135 | 1.11 | 1.14 | 0.79 |
| Cysteine | Cysteine, methionine, SAM, taurine metabolism | C00097 | 1.16 | 1.18 | 0.62 |
| X - 9045 | No Pathway |  | 1.13 | 1.22 | 0.32 |
| Threonine | Glycine, serine and threonine metabolism | C00188 | 1.14 | 1.24 | 0.85 |
| X - 3003 | No Pathway |  | 1.03 | 0.85 | 1.48 |
| Citrulline | Urea cycle; arginine-, proline-, metabolism | C00327 | 1.14 | 1.14 | 0.70 |
| Cysteine-glutathione disulfide | Glutathione metabolism |  | 1.19 | 1.64 | 0.49 |
| X - 6307 | No Pathway |  | 0.73 | 0.93 | 1.55 |
| N-acetylneuraminate | Aminosugars metabolism | C00270 | 1.16 | 0.84 | 1.81 |
| X - 6227 | No Pathway |  | 1.14 | 1.28 | 0.86 |
| Glycocholenate sulfate* | Bile acid metabolism |  | 0.94 | 0.94 | 1.99 |
| Inosine | Purine metabolism, (hypo)xanthine/inosine containing | C00294 | 4.98 | 0.75 | 4.01 |
| X - 02269_201 | No Pathway |  | 1.10 | 1.25 | 0.64 |
| Tryptophan | Tryptophan metabolism | C00078 | 1.07 | 1.06 | 0.78 |
| Mannose | Fructose, mannose, galactose, starch, and sucrose metabolism | C00159 | 0.85 | 0.93 | 1.53 |
| X - 11805 | No Pathway |  | 1.04 | 0.85 | 2.24 |
| 3-carboxy-4-methyl-5-propyl-2-Furanpropanoate (CMPF) | Fatty acid, dicarboxylate |  | 1.58 | 1.68 | 0.77 |
| X - 13543 | No Pathway |  | 0.80 | 0.96 | 0.54 |
| Phenylalanine | Phenylalanine & tyrosine metabolism | C00079 | 1.00 | 0.92 | 1.31 |
| X - 5907 | No Pathway |  | 1.15 | 1.24 | 0.70 |
| Pyroglutamine* | Glutamate metabolism |  | 1.07 | 1.06 | 2.13 |
| Taurocholenate sulfate* | Bile acid metabolism |  | 0.93 | 1.04 | 2.63 |
| X - 8766 | No Pathway |  | 0.89 | 1.06 | 0.65 |
| X - 11469 | No Pathway |  | 1.22 | 1.30 | 0.74 |
| Glutamine | Glutamate metabolism | C00064 | 1.05 | 1.11 | 0.86 |
| X - 14056 | No Pathway |  | 0.69 | 1.16 | 0.47 |
| X - 02249_201 | No Pathway |  | 1.43 | 1.31 | 0.73 |
| Octadecanedioate | Fatty acid, dicarboxylate |  | 0.58 | 0.59 | 1.26 |
| X - 6346 | No Pathway |  | 1.21 | 1.21 | 0.74 |
| Urea | Urea cycle; arginine-, proline-, metabolism | C00086 | 1.14 | 1.22 | 0.88 |
| Gamma-glutamylglutamine | g-glutamyl |  | 0.93 | 1.25 | 0.63 |
| glycylvaline | Dipeptide |  | 1.97 | 0.67 | 2.40 |
| Aspartate | Alanine and aspartate metabolism | C00049 | 1.34 | 0.89 | 1.69 |
| X - 13215 | No Pathway |  | 1.10 | 1.19 | 0.88 |
| Citrate | Krebs cycle | C00158 | 1.16 | 1.11 | 0.86 |
| X - 12456 | No Pathway |  | 0.83 | 0.69 | 1.25 |
| 1-linoleoyl-GPE | Lysolipid |  | 1.09 | 1.50 | 0.82 |
| Hypoxanthine | Purine metabolism, (hypo)xanthine/inosine containing | C00262 | 2.41 | 0.85 | 2.88 |
| Kynurenine | Tryptophan metabolism | C00328 | 0.96 | 0.92 | 1.27 |
| Trans-4-hydroxyproline | Urea cycle; arginine-, proline-, metabolism | C01157 | 1.28 | 1.83 | 0.82 |
| Cortisol | Sterol/Steroid | C00735 | 0.91 | 0.88 | 1.45 |
| X - 9044 | No Pathway |  | 1.03 | 1.15 | 0.67 |
| X - 12850 | No Pathway |  | 1.04 | 1.07 | 2.24 |
| Bilirubin (Z,Z) | Hemoglobin and porphyrin metabolism | C00486 | 1.25 | 1.12 | 0.55 |
| Methionine | Cysteine, methionine, SAM, taurine metabolism | C00073 | 1.05 | 1.06 | 0.91 |
| X - 14658 | No Pathway |  | 0.94 | 0.79 | 2.03 |
| Caprylate (8:0) | Medium-chain fatty acid | C06423 | 1.08 | 1.16 | 0.89 |
