# Supplementary material for: Biomarkers of Inflammation, Immunosuppression and Stress Are Revealed by Metabolomic Profiling of Tuberculosis Patients
Source: PLoS One. 2012 Jul 23;7(7):e40221. doi: 10.1371/journal.pone.0040221 (PMC3402490; doi:10.1371/journal.pone.0040221)
Supplement: Table S4 — Significant clusters of small metabolic compounds containing at least five different members. (DOCX) [file pone.0040221.s007.docx]

1. **Table S4.** Significant clusters of small metabolic compounds containing at least five different members.

| **Cluster** | **N** | **N_full_** | **Examples of fully**  **characterized members** | **Differences between groups*** |
| --- | --- | --- | --- | --- |
| Hormone | 10 | 9 | Androsterone sulfate | N.S. |
| Phenylacetate | 8 | 3 | Phenylacetate, phenylacetylglutamine | N.S. |
| Analgesic/antipyretic xenobiotics | 7 | 7 | Salicylate, 4-  acetaminophen sulfate | N.S. |
| Putative hypoxia-related | 24 | 18 | Inosine, lactate, uridine,  choline | TST^+^/TST^–^, TST^–^/TB*^active^* |
| Carnitine | 9 | 3 | Carnitine, creatine,  piperine | TST^–^/TB*^active^*, TST^+^/TB*^active^* |
| Cholesterol | 11 | 5 | Cholesterol, phosphate,  alpha-tocopherol | TST^–^/TB*^active^*, TST^+^/TB*^active^* |
| Fructose | 6 | 1 | Fructose | TST^–^/TB*^active^*, TST^+^/TB*^active^* |
| Amino acids | 18 | 10 | Tryptophane, citrulline,  proline, threonine,  histidine, inositol-1-  phosphate, glycolate | TST^–^/TB*^active^*, TST^+^/TB*^active^* |
| Kynurenines and taurocholates | 25 | 19 | Phenylalanine, kynurenine, mannose,  taurocholate | TST^–^/TB*^active^*, TST^+^/TB*^active^* |
| Long-chain fatty acids | 35 | 28 | Palmitate, laurate, glycerol, laurylcarnitine | N.S. |
| Fibrinopeptides | 12 | 8 | Fibrinopeptide A, C3f  fragment of the  complement system,  guanosine, leucyl-leucine  (LL) | TST^–^/TB*^active^*, TST^+^/TB*^active^*, TST^+^/TST^-^ |
| Hippurate | 8 | 4 | Hippurate, indoloproprionate | TST^–^/TB*^active^*, TST^+^/TB*^active^* |
| Medium-chain fatty acids | 34 | 22 | Caprylate, isovalerate,  pelargonate | TST^–^/TB*^active^*, TST^+^/TB*^active^* |
| Lysophosphatidylcholines | 30 | 18 | 1-oleoylglycerophosphocholine; bilirubin, biliverdin,  cortisone | TST^–^/TB*^active^*, TST^+^/TB*^active^* |
| Unknown | 6 | 0 | – | N.S. |
| Gamma-tocopherol | 7 | 1 | Gamma tocopherol | N.S. |
| Sulfates | 6 | 2 | 3-indoxyl sulfate, phenol  sulfate | TST^–^/TB*^active^*, TST^+^/TB*^active^* |
| Pyrophosphate | 8 | 3 | 3-methoxytyrosine, pyrophosphate, stachydrine | TST^–^/TB*^active^*, TST^+^/TB*^active^* |

1. Number of members (N); Number of fully characterized members (N_full_); (*****) A cluster is shown to be significantly different between groups if more than two compounds show significant differences between two experimental groups; No significant differences between any two groups (N.S.).
